# Supplementary material for: GI inflammation Increases Sodium-Glucose Cotransporter Sglt1
Source: Int J Mol Sci. 2019 May 23;20(10):2537. doi: 10.3390/ijms20102537 (PMC6566487; doi:10.3390/ijms20102537)
Supplement: Supplementary file 1 [file ijms-20-02537-s001.pdf]

# GI inflammation Increases Sodium-Glucose Cotransporter Sglt1

**Jiyoung Park [1,2,†], In-Seung Lee [1,2], Kang-Hoon Kim [1,2], Yumi Kim [1,2], Eun-Jin An [1,2] and Hyeung-Jin Jang [1,2,*]**

1. College of Korean Medicine, Kyung Hee University, 26, Kyungheedae-ro, Dongdaemun-gu, Seoul, 02447, Republic of Korea; sdu1771@naver.com (J.P.); leejohn1031@khu.ac.kr (I.-S.L.); poklmoo@naver.com (K.-H.K.); yumi0201@khu.ac.kr (Y.K.); aej3866@naver.com (E.-J.A.)
2. Department of Science in Korean Medicine, Graduate School, Kyung Hee University, Seoul 02447, Republic of Korea

* Correspondence: hjjang@khu.ac.kr; Tel: +82-2-961-2315

## Supplementary Materials

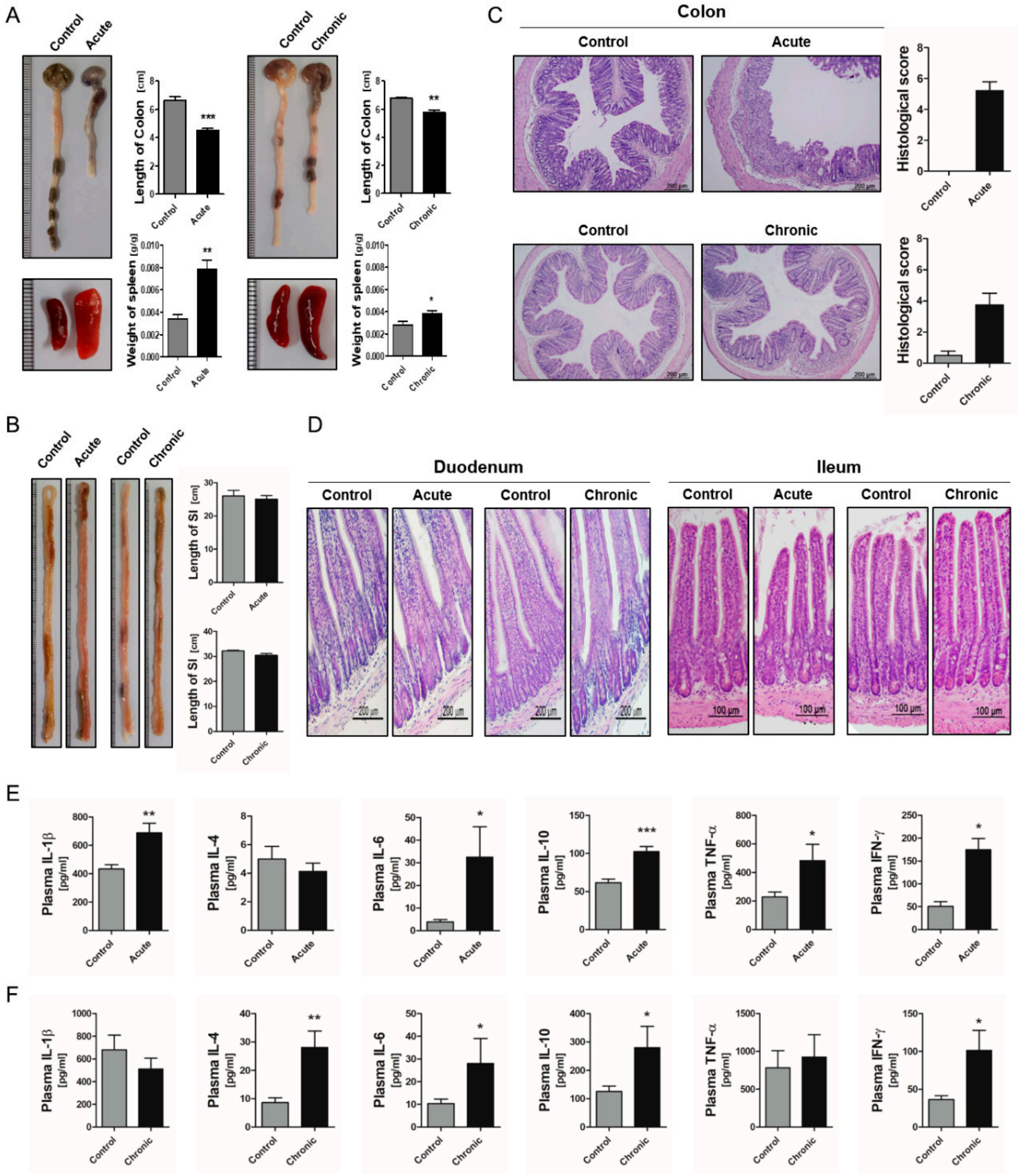


**Supplementary Figure 1. Gastrointestinal tract (GI) inflammatory condition induced by dextran sodium sulfate (DSS) administration.** The index of the GI-inflamed mice. The shortened length of the colon in the acute and chronic groups and the overgrown spleen (right) compared to the control group (left) (A). The small intestine did not show significant changes in its outward appearance (B). In the colon, one-third of the crypts areas was destroyed, and lymphocyte infiltration into the submucosa was observed in the acute group as opposed to the chronic group (C). Scale bar represents 200 μm. The villus of the small intestine was not destroyed by DSS administration and was not directly

modified morphologically (D). In acute group, macrophage-derived Th1 cytokines (IL-1β, IL-6, TNF-α and IFN-γ) and the anti-inflammatory cytokine IL-10 were increased (E). In contrast, cytokines related to the Th2 response were increased in the chronic group (F). Grey bar: Control, Black bar: GI-inflamed mice groups. *$p < 0.05$, **$p < 0.01$, ***$p < 0.001$, Student's *t*-test.

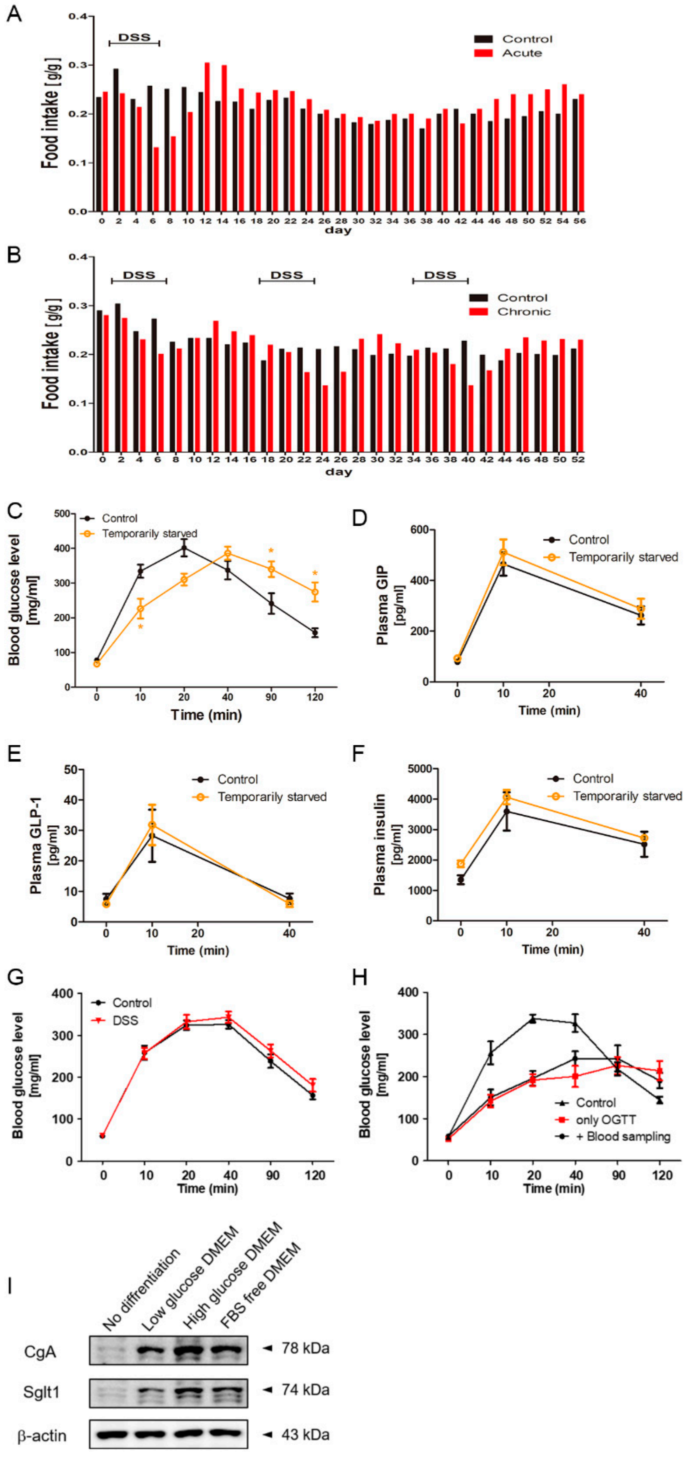


**Supplementary Figure 2. Incretin secretion in response to glucose administration did not affect fasting and blood loss**. Food intake (feed / mice body weight) during recovery in acutely inflamed group (A) and chronic group (B). We provided limited feed to construct a condition similar to decreased food intake in acute group. Compared with starved mice with limited feed for 3 days, during which

dietary intake decreased in acutely inflamed mice. After oral glucose gavage (5 g/kg), blood glucose levels (C), GIP levels (D), GLP-1 levels (E), and insulin levels (F) in fasting mice. n=5/group, $^{*}p < 0.05$, one-way ANOVA. Before DSS administration, the blood glucose level did not differ in the control and GI-inflamed groups (G). The damaged groups were performed only OGTT experiment without blood loss (red square) and with blood loss (circle) compared to the control group (H). No significance between without blood sampling and with blood sampling group. n=10/group. In NCI-H716 cells, differentiation with FBS free media and low glucose DMEM did not increase of Sglt1 protein (I).

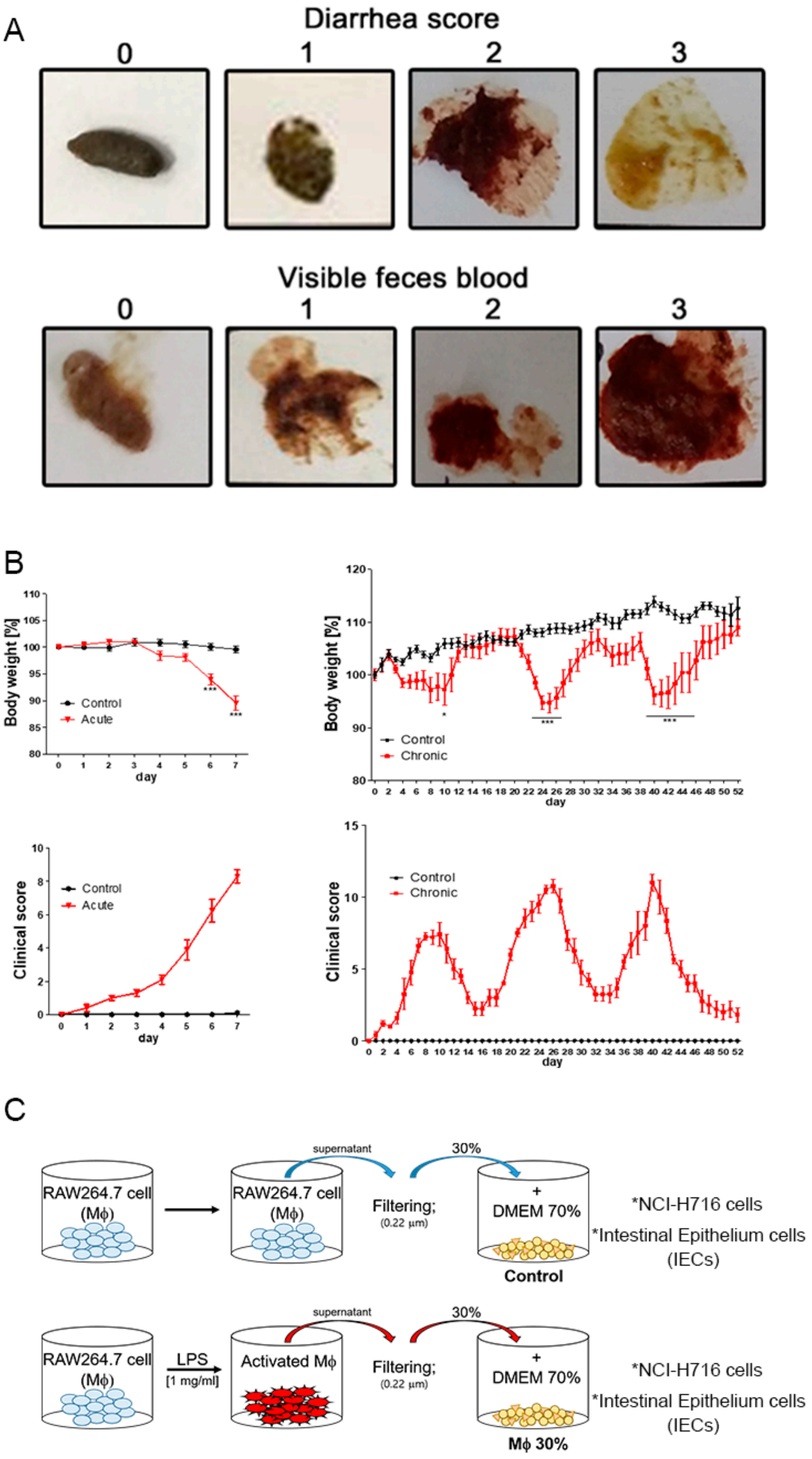


**Supplementary Figure 3. Assessed for the score of diarrhea and feces blood.** For diarrhea score, 0 points were assigned for well-formed pellets, 2 points for pasty and semi-formed stools that did not adhere to the anus, and 3 points for watery stools that did smeared to the anus (A). For visible feces

blood, feces were squashed and scored for the degree of feces blood. Changes in body weight and daily monitoring of the clinical score graph (n=17-20/group) (B). Briefly described summary about preparation of conditioned media (C).

**Supplementary Table 1. Primary antibodies used in immune-reactive experiments**

| Antibodies | Company | Cat. | Dilution range |
|---|---|---|---|
| Sglt1 | Abcam | ab14686 | WB, 1:1000<br>IF, 1:200 |
| GLUT2 | Abcam | ab54460 | WB, 1:1000 |
| T1R2 | Novus Biologicals | NB110-74920 | WB, 1:1000 |
| T1R3 | Bioss antibodies | bs-9113R | WB, 1:1000 |
| Gnat3 | LifeSpan BioSciences | LS-C406431 | WB, 1:1000 |
| β-actin | Santa cruz | sc -47778 | WB, 1:5000 |
| Chromogranin A | Abcam | Ab45179 | WB, 1:1000<br>IHC, 1:200 |
| GLP-1 | Santa Cruz | sc-47778 | IHC, 1:200 |
| GIP | Santa Cruz | sc-57162 | IHC, 1:100 |
| Phosph-ERK | Cell signalling | 9101S | WB, 1:3000 |
| Total-ERK | Cell signalling | 9102S | WB, 1:3000 |
